# Supplementary material for: A Likelihood Approach for Real-Time Calibration of Stochastic Compartmental Epidemic Models
Source: PLoS Comput Biol. 2017 Jan 17;13(1):e1005257. doi: 10.1371/journal.pcbi.1005257 (PMC5240920; doi:10.1371/journal.pcbi.1005257)
Supplement: S1 File — (TAR.GZ) [file pcbi.1005257.s014.tar.gz › HSPH_Online-SI-Revision/MSS10/n1-severe/n1-severe_NEW2_table8.pdf]

1	newly 3 weeks spec	10.	5.06	{0., 15.}	73.8	0.926602	newly 3 weeks cumul	63.	27.91	{5., 50.}	58.7778	0.503102	newly 5 weeks spec	19.	1.62	{0., 5.}	92.1053	1.84558	newly 5 weeks cumul	93.	32.07	{5., 64.}	66.9355	0.62502	newly 46 weeks cumul	179.	33.15	{5., 64.}	81.4804	0.897589
2	newly 3 weeks spec	13.	4.43	{0., 10.}	71.3077	0.976448	newly 3 weeks cumul	51.	29.79	{8., 50.}	45.2745	0.341383	newly 5 weeks spec	4.	1.	{0., 4.}	90.	1.28733	newly 5 weeks cumul	64.	33.18	{8., 60.}	50.3125	0.398089	newly 48 weeks cumul	85.	34.33	{8., 63.}	60.1294	0.50598
3	newly 3 weeks spec	16.	5.42	{0., 17.}	72.25	0.951856	newly 3 weeks cumul	56.	31.1	{9., 58.}	47.6786	0.360766	newly 5 weeks spec	18.	1.14	{0., 5.}	93.6667	1.86499	newly 5 weeks cumul	91.	34.81	{9., 64.}	61.8571	0.523627	newly 47 weeks cumul	131.	35.96	{9., 67.}	72.5496	0.671684
4	newly 3 weeks spec	8.	8.34	{3., 15.}	41.75	0.231815	newly 3 weeks cumul	27.	36.35	{22., 52.}	43.0741	0.152782	newly 5 weeks spec	8.	4.04	{0., 9.}	58.5	0.602497	newly 5 weeks cumul	40.	46.46	{28., 66.}	31.55	0.126005	newly 50 weeks cumul	56.	57.2	{31., 85.}	31.1071	0.141216
5	newly 3 weeks spec	13.	18.2	{8., 28.}	57.3846	0.216735	newly 3 weeks cumul	49.	73.17	{52., 100.}	51.0408	0.168637	newly 5 weeks spec	5.	9.9	{2., 18.}	123.2	0.383583	newly 5 weeks cumul	61.	96.42	{61., 140.}	60.2623	0.1901	newly 49 weeks cumul	65.	126.48	{71., 208.}	96.5231	0.263273
6	newly 3 weeks spec	15.	15.93	{11., 21.}	19.5333	0.0824411	newly 3 weeks cumul	74.	52.47	{43., 61.}	29.1486	0.154027	newly 5 weeks spec	23.	13.82	{8., 19.}	40.4348	0.249776	newly 5 weeks cumul	114.	81.46	{67., 97.}	28.5439	0.150518	newly 46 weeks cumul	183.	172.38	{125., 214.}	15.9563	0.0746625
7	newly 3 weeks spec	8.	15.1	{9., 21.}	91.75	0.272119	newly 3 weeks cumul	25.	55.63	{44., 68.}	124.36	0.353372	newly 5 weeks spec	2.	11.02	{5., 18.}	453.	0.707518	newly 5 weeks cumul	27.	80.28	{62., 99.}	199.185	0.477193	newly 46 weeks cumul	44.	135.28	{94., 174.}	209.364	0.490514
8	newly 3 weeks spec	0.1	3.56	{1., 6.}	3476.	1.38064	newly 3 weeks cumul	0.1	18.39	{11., 25.}	18 290.	2.24115	newly 5 weeks spec	0.1	1.51	{0., 5.}	1496.	0.757776	newly 5 weeks cumul	0.1	22.16	{13., 31.}	22 060.	2.31541	newly 50 weeks cumul	0.1	24.72	{13., 39.}	24 620.	2.35335
9	newly 3 weeks spec	22.	4.	{0., 12.}	84.2727	1.55115	newly 3 weeks cumul	56.	26.57	{5., 57.}	59.1964	0.52412	newly 5 weeks spec	13.	1.03	{0., 1.}	93.	1.91321	newly 5 weeks cumul	88.	30.1	{5., 77.}	68.25	0.680036	newly 46 weeks cumul	139.	32.14	{5., 77.}	77.9424	0.862751
10	newly 3 weeks spec	10.	15.28	{10., 20.}	55.	0.179761	newly 3 weeks cumul	37.	58.12	{48., 66.}	57.0811	0.193236	newly 5 weeks spec	2.	9.63	{5., 14.}	381.5	0.649013	newly 5 weeks cumul	45.	79.29	{69., 89.}	76.2	0.243844	newly 46 weeks cumul	50.	111.18	{92., 134.}	122.36	0.342854
11	newly 3 weeks spec	17.	16.51	{11., 21.}	18.2941	0.0841006	newly 3 weeks cumul	63.	57.18	{47., 66.}	11.6825	0.0554923	newly 5 weeks spec	10.	12.48	{7., 18.}	38.4	0.147342	newly 5 weeks cumul	90.	83.84	{71., 96.}	10.3556	0.0482279	newly 43 weeks cumul	187.	153.9	{121., 186.}	19.4332	0.098461
12	newly 3 weeks spec	0.1	0.88	{0., 3.}	936.	0.318352	newly 3 weeks cumul	10.	9.37	{3., 19.}	53.9	0.276696	newly 5 weeks spec	0.1	0.11	{0., 0.}	202.	0.0538021	newly 5 weeks cumul	10.	9.91	{3., 19.}	58.9	0.286398	newly 51 weeks cumul	10.	9.98	{3., 19.}	59.6	0.287111
13	newly 3 weeks spec	9.	3.13	{0., 8.}	72.7778	0.949889	newly 3 weeks cumul	45.	20.74	{5., 36.}	57.2444	0.46536	newly 5 weeks spec	9.	0.75	{0., 3.}	91.6667	1.62798	newly 5 weeks cumul	69.	23.18	{5., 38.}	67.2464	0.602278	newly 46 weeks cumul	95.	23.92	{5., 40.}	74.8842	0.730268
14	newly 3 weeks spec	8.	7.22	{2., 12.}	43.25	0.258742	newly 3 weeks cumul	35.	32.45	{19., 44.}	21.9714	0.113196	newly 5 weeks spec	7.	3.25	{0., 7.}	67.	0.737169	newly 5 weeks cumul	50.	40.63	{21., 58.}	27.02	0.153129	newly 51 weeks cumul	59.	47.1	{21., 72.}	32.1356	0.183532
15	newly 3 weeks spec	1.	2.11	{0., 7.}	209.	0.750912	newly 3 weeks cumul	11.	12.98	{2., 24.}	61.2727	0.279133	newly 5 weeks spec	1.	0.79	{0., 3.}	151.	0.953202	newly 5 weeks cumul	12.	14.88	{2., 31.}	70.5	0.30708	newly 45 weeks cumul	13.	16.05	{2., 33.}	74.2308	0.320311
16	newly 3 weeks spec	17.	6.9	{1., 13.}	59.6471	0.614417	newly 3 weeks cumul	40.	26.58	{11., 40.}	35.5	0.237184	newly 5 weeks spec	8.	3.59	{0., 8.}	62.875	0.736047	newly 5 weeks cumul	60.	35.82	{13., 59.}	42.1333	0.295742	newly 51 weeks cumul	89.	45.09	{13., 80.}	50.5955	0.384084
17	newly 3 weeks spec	3.	9.19	{4., 14.}	209.667	0.466425	newly 3 weeks cumul	13.	44.16	{32., 56.}	241.692	0.526924	newly 5 weeks spec	2.	3.83	{1., 7.}	123.5	0.40202	newly 5 weeks cumul	18.	53.96	{39., 68.}	202.	0.473879	newly 51 weeks cumul	18.	61.15	{42., 84.}	241.944	0.524715
18	newly 3 weeks spec	24.	8.56	{0., 20.}	68.9167	0.931017	newly 3 weeks cumul	73.	46.16	{10., 85.}	46.0822	0.327877	newly 5 weeks spec	26.	3.53	{0., 11.}	87.8077	1.7169	newly 5 weeks cumul	128.	55.08	{10., 108.}	57.5625	0.479778	newly 51 weeks cumul	297.	58.37	{10., 114.}	80.3468	0.829275
19	newly 3 weeks spec	13.	7.03	{3., 11.}	46.0769	0.335635	newly 3 weeks cumul	52.	27.51	{20., 35.}	47.0962	0.296011	newly 5 weeks spec	8.	3.27	{0., 6.}	62.375	0.617461	newly 5 weeks cumul	69.	36.08	{25., 49.}	47.7101	0.305662	newly 44 weeks cumul	105.	44.05	{26., 62.}	58.0476	0.409065
20	newly 3 weeks spec	5.	5.43	{2., 9.}	49.	0.23863	newly 3 weeks cumul	13.	24.19	{17., 31.}	86.2308	0.257495	newly 5 weeks spec	1.	2.69	{0., 6.}	205.	0.529533	newly 5 weeks cumul	19.	31.14	{21., 42.}	65.0526	0.204834	newly 44 weeks cumul	36.	36.68	{22., 52.}	24.8889	0.10903
21	newly 3 weeks spec	15.	3.34	{0., 9.}	78.1333	1.13875	newly 3 weeks cumul	44.	22.28	{5., 43.}	52.6364	0.429602	newly 5 weeks spec	7.	0.74	{0., 3.}	91.1429	1.5692	newly 5 weeks cumul	62.	24.85	{5., 50.}	60.5	0.530921	newly 51 weeks cumul	100.	25.51	{5., 51.}	74.49	0.729262
22	newly 3 weeks spec	1.	0.93	{0., 2.}	133.	0.794073	newly 3 weeks cumul	13.	9.51	{2., 19.}	49.	0.337912	newly 5 weeks spec	0.1	0.1	{0., 0.}	194.	0.0425527	newly 5 weeks cumul	13.	9.88	{2., 20.}	51.0769	0.340779	newly 45 weeks cumul	13.	9.92	{2., 20.}	51.3846	0.341449
23	newly 3 weeks spec	12.	3.85	{0., 12.}	73.9167	0.987422	newly 3 weeks cumul	38.	21.47	{4., 41.}	52.8158	0.413161	newly 5 weeks spec	6.	0.82	{0., 3.}	88.3333	1.42833	newly 5 weeks cumul	50.	23.97	{4., 46.}	56.78	0.478335	newly 47 weeks cumul	82.	24.88	{4., 47.}	69.9756	0.668508
24	newly 3 weeks spec	7.	1.65	{0., 6.}	80.7143	1.27595	newly 3 weeks cumul	21.	12.36	{2., 25.}	58.381	0.432958	newly 5 weeks spec	2.	0.34	{0., 0.}	105.	1.24632	newly 5 weeks cumul	24.	13.22	{2., 30.}	61.9167	0.47687	newly 44 weeks cumul	30.	13.63	{2., 31.}	65.6333	0.551262
25	newly 3 weeks spec	3.	0.91	{0., 3.}	83.6667	1.07477	newly 3 weeks cumul	19.	7.53	{2., 13.}	61.8421	0.503465	newly 5 weeks spec	2.	0.25	{0., 1.}	94.5	1.17193	newly 5 weeks cumul	27.	8.47	{2., 15.}	69.2963	0.619895	newly 42 weeks cumul	28.	8.73	{2., 15.}	69.4643	0.628817
26	newly 3 weeks spec	6.	4.	{0., 10.}	66.	0.655831	newly 3 weeks cumul	26.	19.51	{6., 36.}	45.4231	0.28779	newly 5 weeks spec	13.	1.45	{0., 4.}	89.7692	1.56333	newly 5 weeks cumul	53.	23.58	{6., 47.}	57.0566	0.466345	newly 49 weeks cumul	100.	26.33	{6., 55.}	73.67	0.707436
27	newly 3 weeks spec	0.1	1.3	{0., 4.}	1332.	0.4951	newly 3 weeks cumul	0.1	7.89	{2., 16.}	7790.	1.75259	newly 5 weeks spec	0.1	0.14	{0., 0.}	230.	0.0698227	newly 5 weeks cumul	0.1	8.29	{2., 17.}	8190.	1.76531	newly 48 weeks cumul	0.1	8.45	{2., 17.}	8350.	1.76901
28	newly 3 weeks spec	18.	8.68	{3., 15.}	51.7778	0.386894	newly 3 weeks cumul	61.	34.02	{22., 45.}	44.2295	0.268275	newly 5 weeks spec	6.	5.38	{2., 10.}	44.	0.276152	newly 5 weeks cumul	78.	46.14	{28., 65.}	40.8462	0.244865	newly 51 weeks cumul	104.	64.46	{38., 95.}	38.5385	0.234702
29	newly 3 weeks spec	6.	6.09	{1., 13.}	60.8333	0.355041	newly 3 weeks cumul	22.	24.73	{11., 40.}	39.8636	0.16902	newly 5 weeks spec	6.	3.24	{0., 8.}	69.	0.735859	newly 5 weeks cumul	37.	32.04	{12., 59.}	38.3784	0.210974	newly 44 weeks cumul	89.	40.47	{12., 74.}	56.6854	0.435361
30	newly 3 weeks spec	5.	5.67	{3., 9.}	42.6	0.1884	newly 3 weeks cumul	19.	20.33	{15., 25.}	16.6842	0.0691473	newly 5 weeks spec	1.	3.42	{1., 7.}	260.	0.53362	newly 5 weeks cumul	23.	27.87	{19., 36.}	26.7391	0.100251	newly 45 weeks cumul	36.	41.46	{27., 58.}	27.6667	0.109067
31	newly 3 weeks spec	5.	13.22	{6., 21.}	169.2	0.414701	newly 3 weeks cumul	36.	50.92	{33., 70.}	46.7778	0.161508	newly 5 weeks spec	7.	8.57	{1., 16.}	59.5714	0.295635	newly 5 weeks cumul	48.	70.29	{44., 94.}	52.6458	0.17873	newly 46 weeks cumul	144.	96.08	{48., 144.}	35.6667	0.220438
32	newly 3 weeks spec	16.	23.16	{14., 32.}	56.375	0.23669	newly 3 weeks cumul	65.	81.09	{67., 95.}	31.0923	0.128894	newly 5 weeks spec	15.	16.34	{9., 24.}	36.4	0.234818	newly 5 weeks cumul	94.	117.49	{94., 143.}	32.6915	0.13915	newly 48 weeks cumul	156.	194.72	{141., 263.}	35.7436	0.15655
33	newly 3 weeks spec	6.	5.17	{1., 11.}	55.5	0.357982	newly 3 weeks cumul	27.	21.07	{9., 33.}	36.1852	0.219736	newly 5 weeks spec	2.	2.9	{0., 7.}	118.	0.611378	newly 5 weeks cumul	33.	27.68	{10., 46.}	42.1212	0.244949	newly 51 weeks cumul	47.	35.85	{11., 68.}	49.0426	0.316142
34	newly 3 weeks spec	10.	3.09	{0., 10.}	75.9	1.16527	newly 3 weeks cumul	29.	18.79	{4., 33.}	49.1379	0.356242	newly 5 weeks spec	9.	0.85	{0., 3.}	91.6667	1.65626	newly 5 weeks cumul	48.	21.53	{4., 42.}	60.3958	0.513311	newly 43 weeks cumul	102.	22.59	{4., 52.}	77.8529	0.812175
35	newly 3 weeks spec	0.1	11.08	{7., 17.}	10 980.	2.01972	newly 3 weeks cumul	3.	39.87	{34., 47.}	1229.	1.11981	newly 5 weeks spec	0.1	6.27	{3., 10.}	6172.	1.74138	newly 5 weeks cumul	3.	54.19	{45., 64.}	1706.33	1.25301	newly 51 weeks cumul	3.	77.23	{60., 100.}	2474.33	1.40288
36	newly 3 weeks spec	0.1	0.93	{0., 3.}	984.	0.338343	newly 3 weeks cumul	0.1	5.9	{1., 14.}	5800.	1.62116	newly 5 weeks spec	0.1	0.14	{0., 0.}	224.	0.0930103	newly 5 weeks cumul	0.1	6.56	{1., 15.}	6460.	1.63944	newly 52 weeks cumul	0.1	6.59	{1., 15.}	6490.	1.63998
37	newly 3 weeks spec	18.	9.71	{4., 16.}	48.2778	0.340377	newly 3 weeks cumul	45.	43.	{32., 56.}	17.2444	0.0791782	newly 5 weeks spec	10.	4.11	{1., 8.}	58.9	0.554186	newly 5 weeks cumul	67.	52.67	{37., 70.}	23.9851	0.126682	newly 42 weeks cumul	100.	62.46	{40., 85.}	37.78	0.221818
38	newly 3 weeks spec	3.	0.83	{0., 2.}	79.6667	1.01867	newly 3 weeks cumul	8.	6.01	{2., 11.}	47.375	0.294753	newly 5 weeks spec	0.1	0.2	{0., 0.}	282.	0.111072	newly 5 weeks cumul	8.	6.74	{2., 14.}	52.25	0.294095	newly 50 weeks cumul	8.	7.03	{2., 14.}	55.625	0.298156
39	newly 3 weeks spec	6.	4.8	{1., 10.}	56.3333	0.425098	newly 3 weeks cumul	26.	19.4	{7., 33.}	37.	0.243398	newly 5 weeks spec	6.	3.23	{0., 8.}	62.8333	0.730477	newly 5 weeks cumul	37.	26.47	{7., 44.}	39.9189	0.276449	newly 46 weeks cumul	74.	37.03	{7., 69.}	53.9865	0.445541
40	newly 3 weeks spec	9.	3.31	{0., 9.}	75.2222	1.01697	newly 3 weeks cumul	26.	20.25	{6., 36.}	48.0385	0.30188	newly 5 weeks spec	4.	0.86	{0., 3.}	96.	1.36319	newly 5 weeks cumul	37.	23.04	{6., 43.}	52.4865	0.386029	newly 52 weeks cumul	38.	23.68	{6., 45.}	53.7368	0.394676
41	newly 3 weeks spec	0.1	7.34	{3., 12.}	7240.	1.81161	newly 3 weeks cumul	9.	30.82	{23., 39.}	242.444	0.525619	newly 5 weeks spec	0.1	3.27	{0., 7.}	3194.	1.30814	newly 5 weeks cumul	9.	39.07	{27., 51.}	334.111	0.626918	newly 49 weeks cumul	9.	46.47	{30., 63.}	416.333	0.69545
42	newly 3 weeks spec	13.	22.57	{1., 37.}	117.615	0.519695	newly 3 weeks cumul	42.	79.94	{18., 116.}	120.19	0.389349	newly 5 weeks spec	12.	15.77	{0., 29.}	89.4167	0.612968	newly 5 weeks cumul	62.	115.32	{20., 170.}	122.355	0.416884	newly 49 weeks cumul	154.	192.44	{21., 288.}	77.3766	0.425236
43	newly 3 weeks spec	2.	3.48	{0., 8.}	135.	0.549368	newly 3 weeks cumul	22.	14.23	{4., 26.}	47.0455	0.337578	newly 5 weeks spec	4.	1.89	{0., 5.}	74.75	0.928511	newly 5 weeks cumul	31.	18.44	{4., 35.}	51.4839	0.393697	newly 42 weeks cumul	85.	22.92	{4., 50.}	73.8588	0.74441
44	newly 3 weeks spec	4.	2.25	{0., 6.}	69.75	0.75611	newly 3 weeks cumul	11.	12.06	{3., 22.}	58.1818	0.278936	newly 5 weeks spec	3.	1.24	{0., 4.}	88.	1.05489	newly 5 weeks cumul	24.	14.86	{3., 29.}	51.1667	0.383322	newly 50 weeks cumul	29.	17.62	{3., 39.}	57.7931	0.440241
45	newly 3 weeks spec	11.	3.64	{0., 8.}	67.4545	0.74975	newly 3 weeks cumul	31.	15.87	{8., 26.}	48.871	0.343787	newly 5 weeks spec	14.	1.57	{0., 4.}	88.7857	1.2734	newly 5 weeks cumul	50.	19.91	{8., 32.}	60.18	0.455557	newly 47 weeks cumul	85.	23.9	{9., 38.}	71.8824	0.618569
46	newly 3 weeks spec	18.	10.32	{3., 18.}	45.6667	0.348624	newly 3 weeks cumul	58.	39.1	{17., 60.}	35.6552	0.225875	newly 5 weeks spec	11.	6.05	{0., 13.}	57.9091	0.555528	newly 5 weeks cumul	91.	52.75	{29., 81.}	42.978	0.283562	newly 46 weeks cumul	196.	72.59	{33., 116.}	63.2398	0.492292
47	newly 3 weeks spec	0.1	1.17	{0., 5.}	1212.	0.43114	newly 3 weeks cumul	0.1	5.77	{1., 12.}	5670.	1.6113	newly 5 weeks spec	0.1	0.07	{0., 0.}	162.	0.0460206	newly 5 weeks cumul	0.1	6.42	{1., 16.}	6320.	1.6328	newly 52 weeks cumul	0.1	6.52	{1., 16.}	6420.	1.63508
48	newly 3 weeks spec	5.	3.97	{0., 9.}	66.2	0.60578	newly 3 weeks cumul	29.	15.83	{5., 30.}	48.7241	0.363773	newly 5 weeks spec	5.	1.84	{0., 6.}	73.2	0.968984	newly 5 weeks cumul	37.	20.91	{7., 38.}	49.7568	0.376782	newly 46 weeks cumul	57.	24.58	{7., 50.}	58.9474	0.501821
49	newly 3 weeks spec	13.	7.	{2., 12.}	49.3846	0.353752	newly 3 weeks cumul	31.	29.86	{20., 39.}	21.5484	0.100539	newly 5 weeks spec	3.	3.47	{0., 7.}	76.3333	0.4847	newly 5 weeks cumul	42.	38.52	{22., 55.}	23.7619	0.117766	newly 50 weeks cumul	46.	45.08	{24., 68.}	29.9565	0.139737
50	newly 3 weeks spec	11.	5.11	{0., 15.}	69.5455	1.0069	newly 3 weeks cumul	42.	27.95	{6., 54.}	47.3571	0.348123	newly 5 weeks spec	10.	2.02	{0., 8.}	90.2	1.54374	newly 5 weeks cumul	58.	32.84	{6., 67.}	51.7241	0.420023	newly 48 weeks cumul	98.	35.4	{6., 71.}	65.3265	0.606281
